# Electrocardiographic Patterns in Patients with Neurally Mediated Syncope

**DOI:** 10.3390/medicina57080808

**Published:** 2021-08-06

**Authors:** Călina-Patricia Țentea, Csilla-Andrea Eötvös, Roxana-Daiana Lazar, Giorgia Paștiu, Iulia-Georgiana Zehan, Mihai Gabriel Andrei, Adriana Porca, Mihaela Jelnean, Roxana Mihaela Chiorescu, Larisa-Diana Mocan-Hognogi, Sorin Pop, Dan Blendea

**Affiliations:** 1 Cluj County Emergency Hospital, 400000 Cluj-Napoca, Romania; tenteapatricia@yahoo.it (C.-P.Ț.); daiana.pocol@yahoo.com (R.-D.L.); giorgia23pastiu@yahoo.com (G.P.); iuliazehan@gmail.com (I.-G.Z.); gandrei_93@yahoo.com (M.G.A.); adriana_porca@yahoo.com (A.P.); mihaelajelnean@gmail.com (M.J.); roxi_11_77@yahoo.com (R.M.C.); dyi_larisa@yahoo.com (L.-D.M.-H.); popsorin98@gmail.com (S.P.); 2 Department of Medicine, Faculty of Medicine, University of Medicine and Pharmacy “Iuliu Hatieganu”, 400012 Cluj-Napoca, Romania; csilla.andrea18@gmail.com; 3 “Niculae Stancioiu” Heart Institute, 400001 Cluj-Napoca, Romania

**Keywords:** neurally mediated syncope, electrocardiogram, cardioinhibitory reflex

## Abstract

The baseline electrocardiogram (ECG) is less informative in neurally mediated syncope (NMS) than in arrhythmic syncope. However, some of the ECG patterns present in NMS can have diagnostic and prognostic value in such patients. Electrocardiographic documentation of a syncopal spell and thus identification of the ECG changes can be performed during tilt table test (TTT) or during prolonged ECG monitoring. This work reviews the specific ECG patterns in NMS, which are primarily related to the cardioinhibitory reflex. In addition, there are other ECG findings present in patients with NMS that are being analyzed, such as increased heart rate variability as well as specific QRS voltage patterns. In addition to the diagnostic and prognostic value, these ECG patterns in NMS may help improving the selection of patients for pacemaker implant.

## 1. Introduction

Given that syncope is an entity frequently seen in the emergency department, it is essential for clinicians to distinguish between neurally mediated syncope (NMS) and other causes of transient loss of consciousness (TLOC), in order to establish an accurate diagnosis and provide appropriate medical treatment. While determining the cause of syncope relies significantly on clinical findings, the electrocardiogram (ECG) can add valuable information.

In the 2017 American College of Cardiology/American Heart Association/Heart Rhythm Society (ACC/AHA/HRS) syncope guideline, the ECG has a class I recommendation, together with history and physical exam as part of the initial evaluation, during which it can help identifying an underlying arrhythmogenic substrate for the syncopal episode [1]. The ECG can also have characteristic patterns in Wolff–Parkinson–White syndrome, Brugada syndrome, long-QT syndrome, hypertrophic cardiomyopathy, or arrhythmogenic right ventricular cardiomyopathy, which can all be associated with syncope [1].

The baseline ECG is perhaps less informative in NMS than in arrhythmic syncope. However, it can still offer some patterns, more or less specific to NMS, that could help with the diagnosis and prognosis in such patients. Most of the time, patients with NMS are asymptomatic at the time of the clinical examination, and the likelihood of capturing a spontaneous event is low. Documentation of a syncopal spell, and thus clarification of the mechanism, relies on tilt table test (TTT) and prolonged ECG monitoring.

Specific ECG patterns in NMS are primarily related to the cardioinhibitory (CI) reflex. In addition, there are other ECG findings such as increased heart rate variability as well as specific QRS voltage patterns that could that have clinical importance in patients with NMS.

The purpose of this work is to review the different ECG patterns that can occur in patients with NMS and to describe the value of the ECG in the management of such patients.

## 2. Pathophysiology of Neurally Mediated Syncope

Syncope is defined as a sudden, short-duration TLOC, which occurs in the context of inadequate cerebral perfusion [2]. The main mechanism in NMS is represented by an abnormal autonomic response initiated by diverse triggers, such as prolonged standing, strong emotions, cough, micturition, or defecation [3]. The triggers activate peripheral or central receptors and the vagal central sites, which have a pivotal role in the reflex mechanism of NMS, responding to stimulus with an efferent CI and/or a vasodepressor component [4,5,6]. The response to these triggers is more likely to occur or to be more severe when hypovolemia, hypoxemia, or thermal stress are present [2]. Knowing that cerebral perfusion is mainly dependent on systemic blood pressure (BP), and that BP depends on stroke volume, heart rate (HR), and peripheral vascular resistance, syncope can appear whenever one of these parameters decreases, without being efficiently compensated by an increase of any of the other two [3,7]. 

The afferent neural signals are mainly transmitted to the nucleus solitarius, which has multiple connections with the dorsal and ambiguous nuclei of the vagus nerve, usually triggering two types of responses. The first one results in generating a defense reaction that includes a sympathetic neural activation. On the opposite hand, the second one is characterized by sympathetic withdrawal and parasympathetic activation. The overactivation of the latter mechanism is responsible for vasodilation, bradycardia, and consequent syncope [8]. It is known that the mechanism of NMS also involves disturbances in different serum neurohormonal levels. These include adrenaline, noradrenaline, serotonin, nitric oxide, and pancreatic polypeptide [3]. The interaction between neural activation and humoral factors influences the manner in which a syncopal event becomes manifest.

**Figure 1:**
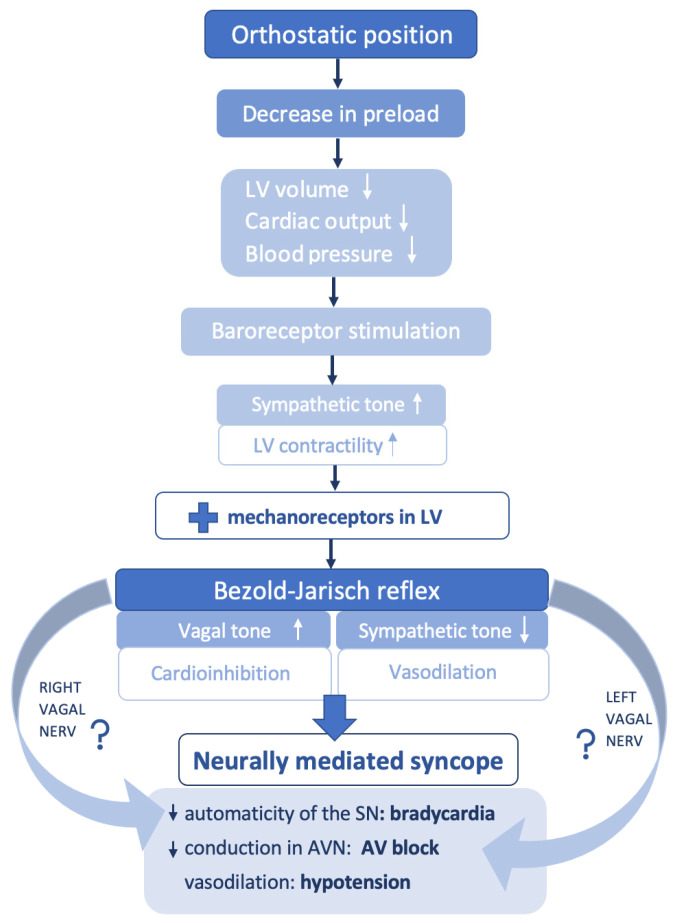
Pathophysiology in neurally mediated syncope. LV = left ventricle, SN = sinus note, AV = atrioventricular, AVN = atrioventricular node.

In addition, studies on postural syncope revealed a potential involvement of the cardiac mechanoreceptors in mediating the vagal response [7]. Normally, due to blood pooling in the lower extremities while standing, the circulating volume drops and BP decreases. Signals are further transmitted to the central sites in order to generate a compensatory reaction and to avoid cerebral hypoperfusion. Therefore, sympathetic stimulation leads to an increase of the peripheral resistance along with positive chronotropism and inotropism [7]. As people with NMS tend to have exaggerated venous pooling while standing, a forceful heart contraction on an underfilled ventricle will stimulate the cardiac mechanoreceptors with vagal afferent pathway and will trigger an inhibitory response (Bezold–Jarisch reflex), with consequent bradycardia, vasodilatation, hypotension, and TLOC (Figure 1). This concept is known as the ventricular theory, and it is widely accepted [7].

The described hemodynamic mechanisms vary with different factors, one of them being age, with younger patients being more prone to becoming asystolic during vagal overstimulation [9].

## 3. Cardioinhibitory Response

**Figure 2:**
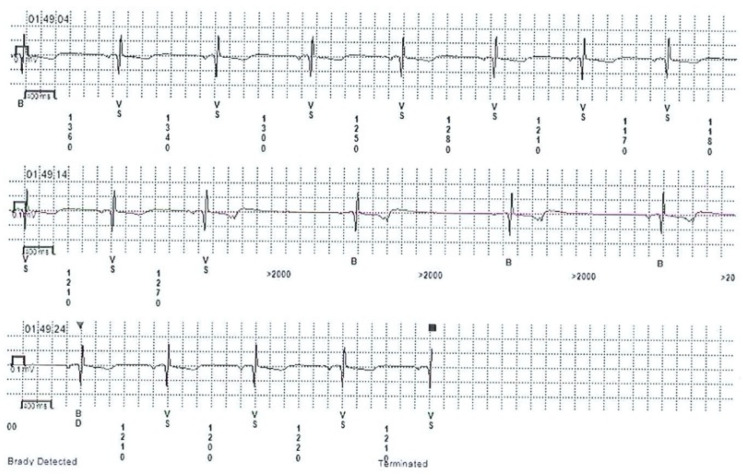
Implantable loop recorder tracing revealing cardioinhibitory response during presyncope in a patient with neurally mediated syncope.

Cardioinhibitory response was previously referred to as sinoatrial syncope or malignant vasovagal syndrome [10]. There are limited data in the literature regarding ECG findings in CI syncope, as it encompasses a relatively small group of patients with NMS. The clinical relevance lies within its trend to affect predominantly young people, presenting with atypical recurrent syncope and less pronounced prodromal symptoms. ECG manifestations of the CI response can be detected incidentally on telemetry in the emergency department, or during hospitalization in patients with spontaneous syncope. The CI response can also be documented using heart rhythm monitoring devices such as a Holter monitors, event monitors, or implantable loop recorders (Figure 2). Induced CI response is less frequent than the spontaneous response, being present in only 28% of the TTT-induced NMS, while spontaneous CI was encountered in 53% of cases [11,12,13].

**Figure 3:**
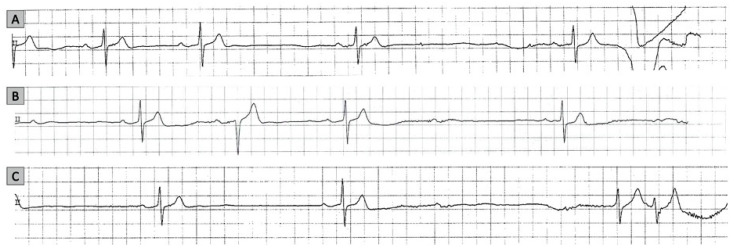
Cardioinhibitory response recorded on telemetry in a patient with neurally mediated syncope. Progressive slowing of sinus rate and first-degree atrioventricular (AV) block (panel (**A**)), and two episodes of second-degree AV block (panels (**B**,**C**)). The association of sinus bradycardia and different types of AV block is highly suggestive for a vagal mechanism for syncope.

The CI response due to a reflex mechanism is typically a diagnosis of exclusion, after the anatomical involvement of the sinus node (SN) and atrioventricular (AV) node was ruled out. A concordant depression of both SN and AV node function is suggestive of a vagally mediated mechanism [11,14] (Figure 3). 

A decrease in HR with more than 30% of baseline and less than 40 bpm for a period longer than 10 s or progressive/initial sinus bradycardia before sinus arrest are common expressions of SN inhibition. Sinus arrest results in asystole, with no atrial or ventricular electrical activity recorded for at least 3 s. Sinus arrest without escape rhythm lasting more than 3 s after a blocked P-wave might be present as well [12,13].

The AV node depression in NMS has heterogeneous presentation, including several subtypes of AV block, such as second-degree AV block type I or type II, 2:1 AV block, high-grade AV block, and third-degree AV block, or, more important, the association of two or more subtypes [11].

The ECG patterns that can result from different degrees of inhibition of SN and AV node are variable. These patterns occur with different frequencies depending on whether the syncope is induced (during TTT) or spontaneous. The differences are apparent when comparing data on induced syncope observed during TTT in the study of Zysco et al. [13] and during spontaneous syncope diagnosed as likely neurally mediated with data documented by implantable loop recorder (ILR) in the ISSUE 2 study [11,12] and the study of Brignole et al. [15]. Sinus arrest alone occurred in 23% of patients with induced vs. 30% of patients with spontaneous syncope. SN inhibition with sinus arrest during spontaneous syncope seems to be present equally in young patients and in the elderly. In contrast, induced syncope with sinus arrest during TTT tends to affect younger patients more than older patients [12]. Another distinct pattern is that of AV block with sinus bradycardia or arrest, which occurred in 5% of induced and 9% of spontaneous syncope. Atrioventricular block alone was found to be extremely rare in patients with induced syncope and in approximately 15% of patients with spontaneous syncope [11,12,13].

Sinus arrest or sinoatrial block in NMS is more frequent than AV block, either because of a higher sensibility to vagal stimulation than the AV node or because vagal stimulation of the SN with marked sinus bradycardia and pauses does not allow the AV block to manifest itself on the ECG due to lack of any atrial electrical activity [11]. Given the last observation, neurally mediated AV block could be a more underdiagnosed phenomenon rather than a rare one.

Zysko and colleagues [13], when studying 31 patients who had TTT-induced AV block, found the association of at least two types of AV block in the same patient. For example, type I second degree AV block (present in 35.5% of patients) was present at the beginning and was followed by advanced AV block (present in 67.8% patients), ventricular asystole with non-conducted P-waves, ending with atrial and ventricular asystole; the most common escape rhythm was junctional rhythm present in 61.3%. A 2:1 second-degree AV block and third-degree AV block were found as well, with a prevalence of 48.4% and 41.9%, respectively. It is worth mentioning that the majority of the patients had an association of at least two types of AV block during TTT. Interestingly, in patients who developed AV block provoked by TTT, the timing of syncope was the test termination/ending point, as well as during the recovery period, or both.

In another study, Alboni et al. [14] noted a higher frequency of vagally mediated AV block episodes during the night, in the absence of sleep disorders.

Considering evidence from animal studies that SN is more sensitive to right vagus nerve stimulation, and AV node to the left vagus nerve stimulation [10,16], patients with NMS and AV block could have a preferentially left branch activation, whereas those with sinus arrest, a preferred right branch activation (Figure 1). Consequently, this hypothesis could help in understanding the lack in uniformity of events in NMS (as NMS is associated either with sinus arrest, AV block, or a combination of the two).

Avbelj et al. [17] revealed another interesting electrocardiographic pattern using multichannel ECG measurements in NMS with CI response. The study confirmed a sudden change in the morphology of the P-wave alongside a shortening of the PQ interval. Given the conduction velocity in the right atrium walls is around 88 cm/s, it would require a 2 cm shift of the main pacemaker towards the AV node in order to explain both the change in morphology of the P-wave and the shortening of the PQ interval [17]. This observation provides new insight in matter of physiological mechanisms implicated during CI process.

**Figure 4:**
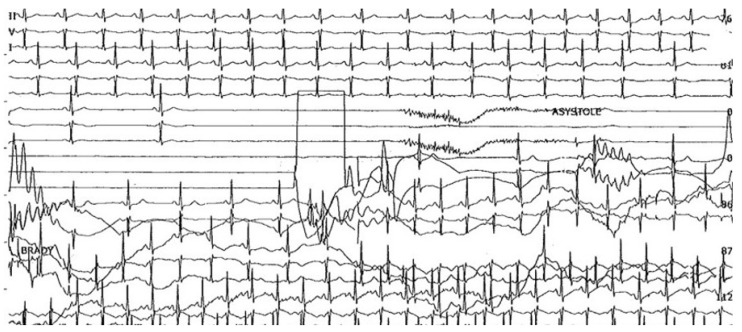
Example of cardioinhibitory response captured on electrocardiogram in neurally mediated syncope: the inhibitory effect of vagus stimulation on the sinus node results in progressive bradycardia, followed by asystole without P-waves and acceleration of the sinus rhythm in the second part of the tracing.

It is worth noting the uncommon presentation forms of NMS, such as atrial fibrillation and supraventricular tachycardia. A few studies mention a minority of patients who had atrial tachyarrhythmia after asystole [10], or atrial flutter with AV block during TTT followed by syncope [18]. Although it is more common for sinus tachycardia to succeed the asystolic episode during syncope (Figure 4), there have been cases in which it preceded the asystolic episode, accompanied by a significant drop in the mean BP, suggesting a vasodepressive component [19,20].

Although CI response during NMS may have dramatic consequences, the reaction is usually self-limited and not life-threatening.

## 4. The Importance of the Cardioinhibitory Reflex for Patient Selection for Pacemaker Implantation

Identifying the CI reflex in TLOC patients is important when making the decision to implant a pacemaker. This reflex can be induced at the time of TTT or can be spontaneous when is detected in real life during ECG monitoring. The treatment in NMS patients can vary from educational and drug interventions to pacemaker implantation. Proper selection of patients is the key when choosing patients who will benefit the most from pacing, but we require further studies to establish such pertinent criteria [1].

TTT can be used to unmask NMS, and the results can predict response of cardiac pacing in NMS [21,22]. Asystolic tilt response shows the major role of CI reflex in NMS and predicts asystolic spontaneous syncope, and thus the response to DDD-CLS (closed loop stimulation) pacing in reducing the syncope burden and syncope recurrence [23,24,25,26,27]. It was observed that in patients with NMS, in spite of DDD-CLS stimulation, prodromal symptoms were still present. When prodromal symptoms were followed by syncope, the mean time between the onset of symptoms and the occurrence of syncope was significantly longer in DDD-CLS mode compared with DDD mode [27]. Quality of life improvement was noticed in these patients [28].

Temporal sequence of events in NMS with asystole may show the ideal candidate for CLS pacing: patients in whom asystole occurs before syncope, with BP being normal or mildly decreased in this phase [29]. Video recording and electroencephalographic monitoring in TTT can help identifying these patients, considering asystole as the main mechanism when asystole and syncope occur in close proximity of each other [30,31]. Thus, the importance of proper delineation in identifying patients in whom overlap of vasodepression and cardioinhibition is present during syncope. [30,31]. 

Saal et al. [30] showed that in asystolic NMS, electrocardiographic monitoring alone used for documenting asystole can overestimate the importance of asystole. In one-third of cases asystole occurred too late to be the main cause of NMS. This phenomenon may be the explanation for pacing response failures noted in some studies.

Although the majority of NMS are benign, there are some cases which may benefit from cardiac pacing, such as recurrent and severe NMS and confirmed asystolic episode, in spite of conservative management, according to ACC/AHA/HRS 2017 guidelines [1,32]. Current European guidelines recommend pacing due to asystole, AV block, or a combination of the two, in patients over 40 years of age presenting spontaneous symptomatic pause longer than 3 s or asymptomatic pause longer than 6 s [2,32]. However, there are no data supporting cardiac pacing in NMS patients younger than 40. It is worth mentioning that the implantation of a pacemaker will not be beneficial in the absence of a documented bradycardia-induced syncope due to vasovagal reflex [25,32,33].

## 5. Heart Rate Variability

Since the orthostatic position can play a role in the pathogenesis of NMS, TTT is commonly used to gather information about the mechanism of syncopal episodes using rhythm and blood pressure monitoring [34].

Previous research that analyzed the variation of cardiac parameters including the heart rate (HR) in patients with NMS undergoing TTT identified four phases of the syncopal episode: early stabilization, circulatory instability, terminal hypotension, and recovery [5].

During the phase of circulatory instability, there is a fall in systolic blood pressure and cardiac output, and onset of increased BP variability [5]. These changes appear to be associated in some patients with positive TTT with an increase in heart rate variability (HRV) [35]. Several studies using spectral analysis of the HR evidenced an increase in the low-frequency (LF) component at rest and an increase in the LF to high-frequency (HF) ratio before the syncopal event during TTT [10]. In a case–control observational study, Miranda et al., who compared patients with CI vasovagal syncope with controls with no syncope and negative TTT, showed that the HRV components could predict the CI response before beginning the tilting, and some of them, when compared in the different phases of TTT, differed from those in the control group [36]. The LF component in the supine position showed sensitivity of 97.4% and specificity of 83.3% in detecting patients with history of syncope, with area under the curve (AUC) 0.75) [36].

The HR itself seems to be higher in patients with NMS who will develop a positive TTT. Mallat and colleagues [35] evaluated the early increase of the HR during TTT in patients with recurrent syncope and showed that an increase of HR less than 18 bpm in the first 6 min of the testing was highly predictive for a negative test.

Heart rate variability has been evaluated in patients with NMS as a surrogate for reinnervation after cardioneuroablation (CNA). Pachon and colleagues [37] found that minimum and mean HR increased significantly at two years after CNA, but between the first and the second years, there were no essential changes, emphasizing the idea that no reinnervation occurred [38]. In addition, CNA eliminates the CI reflex and keeps most patients asymptomatic, maintaining exercise tolerance and also preventing pacemaker implantation [39]. This result is offering an encouraging treatment option for patients with NMS [37,40,41].

Akizuki et al. examined the autonomic activity of patients with NMS presenting after a syncopal episode to the emergency department. They used HRV measurements during an orthostatic test and found that both LF and HF decreased, and HR increased after syncope, while the coefficient of variation of the RR interval on ECG was significantly decreased in patients with NMS [42]. Both LF expressed in normalized units and the LF/HF ratio increased on standing, and these changes were accompanied by a decrease in normalized HF in both groups, consistent with sympathetic activation and parasympathetic withdrawal in the heart [42].

## 6. QRS Voltage

**Figure 5:**
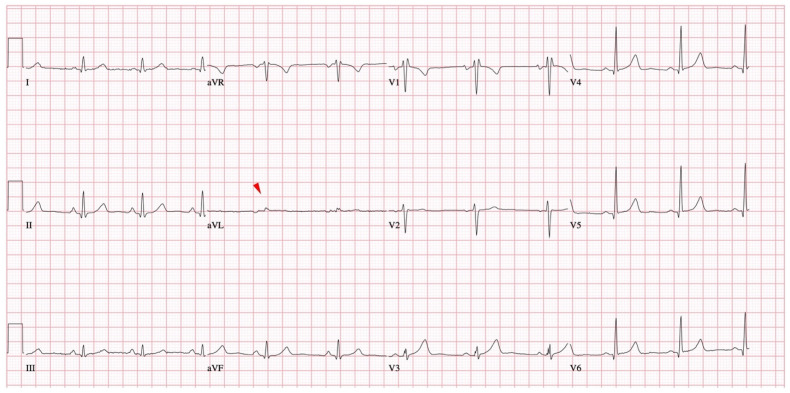
Isolated very low QRS voltage in frontal plane leads (red arrowhead).

A recently studied ECG pattern related to NMS is the presence of isolated very low voltage (VLV) QRS complexes. VLV was defined as a voltage of ≤0.3 mV in frontal leads and of ≤0.7 mV in precordial leads. These aspects were first described starting from an empirical observation that some patients with NMS have low voltage QRS complex present in an isolated frontal ECG lead [43] (Figure 5).

This ECG pattern appears different from the usual low voltage, which is defined as a QRS amplitude of less than 0.5 mV in all frontal leads and less than 1 mV in all precordial leads, and which is usually due to a cause that affects voltage generation or voltage transmission from the heart to the surface electrodes [44].

In a study that included 216 patients with suspected NMS and who underwent a TTT, as part of their diagnostic workup, the lowest QRS amplitude present in the frontal leads was most frequently encountered in lead aVL. The lead displaying VLV was close to perpendicular to the QRS axis. The voltage in frontal leads was found to be significantly lower in the TTT-positive group of patients in comparison with the TTT-negative group [43]. 

The mechanism for this isolated QRS voltage reduction is not known. However, given the fact that there was a significant correlation between the QRS with lowest voltage and LV end-diastolic diameter, the authors hypothesized that the presence of isolated VLV is due at least in part to a specific LV geometry encountered in patients with NMS [43].

The relationship between QRS voltage and intracardiac and intravascular volume was first described by Brody, who found that an increase in intracavitary blood mass increases cardiac forces transmission by decreasing impedance between the heart and extracardiac tissue [45]. Therefore, the amplitude of the electrical activity generated by the ventricle will be inscribed on the surface ECG without reduction of voltage. On the other side, low blood volume would generate a low amplitude QRS by means of increasing blood tissue impedance. Low intravascular volume and small cardiac dimensions are known to be involved in the pathogenesis of NMS [46,47].

These data could be integrated in a mechanistic hypothesis. The frontal lead that displays the VLV is nearly perpendicular to the QRS axis. Cancellation of electrical forces in the ventricles in a direction that is perpendicular to the QRS axis might generate low voltage. Opposing left ventricular walls may be in closer proximity in patients with NMS because of ventricular underfilling, and this could accentuate the phenomenon of cancelation along the short axis of the ventricle. Thus, increased cancellation and the resulting reduction in voltage along the short axis may generate isolated low QRS voltage in patients with NMS [48].

Isolated VLV was found to have predictive value for recurrence of syncope in patients with NMS. In a cohort of patients with suspected NMS, with a median of three syncopal episodes, VLV in frontal leads was present in 45% of the patients. After a median follow-up period of 14 months, the actuarial total syncope recurrence rate was 22% at 1 year. Patients with isolated VLV in frontal leads demonstrated threefold increase of the risk of recurrent syncope, compared to those without isolated VLV, independent of clinical factors that predict recurrence of syncope [48]. Such risk factors included the number of ≥2 episodes of syncope [49]. It is of note that smaller end-diastolic LV dimensions were also predictive of syncope recurrence in the multivariate model, underlining the importance of low intravascular volume in the pathogenesis of NMS. One interesting finding of this study was that at baseline, the presence of VLV seemed to be associated with vasodepressor and not CI response on TTT. Vasovagal Syncope International Study (VASIS) type 1 (mixed response) and type 3 (vasodepressor) responses to TTT were significantly more frequent in patients with VLV when compared to patients without VLV in frontal leads. No significant differences between patients with VLV and without VLV were found for the VASIS response type 2 (cardioinhibitory) [48]. 

Patients with NMS have reductions in voltage not only in frontal leads, but also in the precordial leads [43,48]. VLV in precordial leads, defined as a QRS voltage of ≤ 0.7 mV, was present in 36% of the 135 patients with NMS enrolled in a prospective study, with lead V1 displaying this pattern in most of the cases. After a median follow-up of 15 months, syncope recurrence rate was 26% in patients with VLV in precordial leads and 21% in patients without VLV. The precordial VLV was found to be predictive independently of VLV in frontal leads [50].

The proposed alteration of ventricular activation, geometry, and associated isolated VLV was further studied by vectorcardiography, thus providing a better spatial display of the magnitude and direction of the activation vectors. 

**Figure 6:**
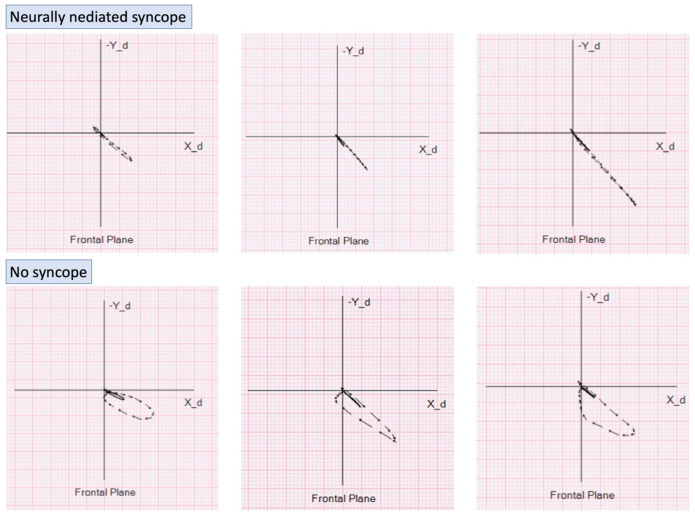
Examples of flat vectorcardiographic loops in the frontal plane in three patients with neurally mediated syncope—top panel. Vectorcardiographic loops in the frontal plane in three subjects with no history of syncope—bottom panel.

Preliminary research suggests that QRS loop in the frontal plane are smaller and more elongated in patients with NMS [51] (Figure 6). When compared to a syncope-free population and without significant cardiac abnormalities, the QRS loop area and width-to-length ratio in NMS patients were significantly lower. This finding was also shown to have predictive value. In a prospective study of 190 patients, followed for a median of 10 months, the presence of flat QRS loops in frontal plane leads was found to predict recurrence of NMS independent of isolated VLV and LV end-diastolic diameter [52]. 

Given that these ECG and vectorcardiogram (VCG) parameters were strongly predictive of recurrent syncope, an attempt was made to incorporate them in a predictive model. This model included risk factors according to their relative importance, inferred from the hazard ratios of the Cox multivariate analysis, which identified history of ≥2 syncopal events, LV end-diastolic diameter of <39 mm by echocardiography, isolated VLV QRS in frontal leads, and flat QRS VCG loops in frontal plane as independent predictors for NMS recurrence. Three points were assigned for history of isolated VLV in frontal leads and ≥2 syncopal events, two points for flat QRS VCG loops in frontal plane, and one point for LV end-diastolic diameter of <39 mm measured by echocardiography. The total risk score included three categories: low risk (0–2), intermediate risk (3–5), and high risk (≥6). The actuarial total syncope recurrence rate at 1 year was 54.6% in the high-risk score category, 25.3% in the intermediate risk category, and 6.2% in the low-risk category. The receiver operating characteristic (ROC) curve showed an AUC of 0.77 for the predictive value of the total risk score [53].

To summarize, the presence of isolated VLV in frontal leads is a parameter that could be applied in the workup of patients with syncope: it could help in the process of clarifying the diagnosis when the clinical variables do not offer sufficient clarity, and most importantly it could help stratify the risk of recurrence of patients with NMS. The latter could potentially be used in the process of selecting patients for pacemaker implantation. However, this would require validation in randomized prospective trials.

## 7. Summary and Conclusions

The ECG remains crucial for identifying the etiology arrhythmic syncope and can also offer important clues to possible NMS. Specific ECG patterns in NMS are primarily related to the CI reflex. The ECG manifestations of the CI reflex are variable and result from different degrees of inhibition of SN and AV node. Sinus arrest preceded by progressive sinus slowing and followed by progressive sinus acceleration is one of the most frequent ECG patterns. Sinus arrest without escape after a blocked P-wave might be present as well. The AV node depression in NMS results in several subtypes of AV block: second degree AV block type I or type II, 2:1 AV block, high-grade AV block, and third-degree AV block.

Increased HRV is another ECG pattern that was identified in patients with NMS undergoing TTT, especially during the phase of circulatory instability. This pattern was found to be predictive of a CI reflex in certain patients.

Isolated very low QRS voltage on frontal ECG leads were recently described to predict a positive TTT and recurrent syncope in patients with NMS. Isolated VLVs are more prevalent in patients with small dimensions of the LV on echo. Preliminary results suggest that this ECG finding is associated predominantly to a predisposition for vasodepressor or mixed response. Whether this novel ECG predictor relates in any way to the CI response as well remains to be clarified in future research. This could have importance to improve selection of NMS patients for pacemaker implantation.

## References

[1] Shen W.K., Sheldon R.S., Benditt D.G., Cohen M.I., Forman D.E., Goldberger Z.D., Grubb B.P., Hamdan M.H., Krahn A.D., Link M.S. (2017). 2017 ACC/AHA/HRS Guideline for the Evaluation and Management of Patients with Syncope: A Report of the American College of Cardiology/American Heart Association Task Force on Clinical Practice Guidelines and the Heart Rhythm Society. Circulation.

[2] Brignole M., Moya A., de Lange F.J., Deharo J.C., Elliott P.M., Fanciulli A., Fedorowski A., Furlan R., Kenny R.A., Martin A. (2018). 2018 ESC Guidelines for the diagnosis and management of syncope. Eur. Heart J..

[3] van Dijk J.G., van Rossum I.A., Thijs R.D. (2020). Timing of Circulatory and Neurological Events in Syncope. Front. Cardiovasc. Med..

[4] Rocha B.M.L., Gomes R.V., Cunha G.J.L., Silva B.M.V., Pocinho R., Morais R., Araujo I., Fonseca C. (2019). Diagnostic and therapeutic approach to cardioinhibitory reflex syncope: A complex and controversial issue. Rev. Port. Cardiol..

[5] Jardine D.L., Wieling W., Brignole M., Lenders J.W.M., Sutton R., Stewart J. (2018). The pathophysiology of the vasovagal response. Heart Rhythm.

[6] Wieling W., Jardine D.L., de Lange F.J., Brignole M., Nielsen H.B., Stewart J., Sutton R. (2016). Cardiac output and vasodilation in the vasovagal response: An analysis of the classic papers. Heart Rhythm.

[7] Mosqueda-Garcia R., Furlan R., Tank J., Fernandez-Violante R. (2000). The elusive pathophysiology of neurally mediated syncope. Circulation.

[8] Benditt D.G. (1997). Neurally mediated syncopal syndromes: Pathophysiological concepts and clinical evaluation. Pacing Clin. Electrophysiol..

[9] Stewart J.M., Medow M.S., Sutton R., Visintainer P., Jardine D.L., Wieling W. (2017). Mechanisms of Vasovagal Syncope in the Young: Reduced Systemic Vascular Resistance Versus Reduced Cardiac Output. J. Am. Heart Assoc..

[10] Mehlsen J., Kaijer M.N., Mehlsen A.B. (2008). Autonomic and electrocardiographic changes in cardioinhibitory syncope. Europace.

[11] Brignole M. (2009). Different electrocardiographic manifestations of the cardioinhibitory vasovagal reflex. Europace.

[12] Brignole M., Sutton R., Menozzi C., Garcia-Civera R., Moya A., Wieling W., Andresen D., Benditt D.G., Grovale N., De Santo T. (2006). Lack of correlation between the responses to tilt testing and adenosine triphosphate test and the mechanism of spontaneous neurally mediated syncope. Eur. Heart J..

[13] Zysko D., Gajek J., Kozluk E., Mazurek W. (2009). Electrocardiographic characteristics of atrioventricular block induced by tilt testing. Europace.

[14] Alboni P., Holz A., Brignole M. (2013). Vagally mediated atrioventricular block: Pathophysiology and diagnosis. Heart.

[15] Brignole M., Gaggioli G., Menozzi C., Del Rosso A., Costa S., Bartoletti A., Bottoni N., Lolli G. (2000). Clinical features of adenosine sensitive syncope and tilt induced vasovagal syncope. Heart.

[16] Schiereck P., Sanna N., Mosterd W.L. (2000). AV blocking due to asynchronous vagal stimulation in rats. Am. J. Physiol. Heart Circ. Physiol..

[17] Avbelj V., Trobec R. (2013). A closer look at electrocardiographic P waves before and during spontaneous cardioinhibitory syncope. Int. J. Cardiol..

[18] Chou H.H., Lin K.H., Luqman N., Kuo C.T. (2003). Prolonged ventricular asystole, sinus arrest, and paroxysmal atrial flutter-fibrillation: An uncommon presentation of vasovagal syncope. Pacing Clin. Electrophysiol..

[19] Leitch J.W., Klein G.J., Yee R., Leather R.A., Kim Y.H. (1992). Syncope associated with supraventricular tachycardia. An expression of tachycardia rate or vasomotor response?. Circulation.

[20] Moya A., Brignole M., Menozzi C., Garcia-Civera R., Tognarini S., Mont L., Botto G., Giada F., Cornacchia D., on behalf of the International Study on Syncope of Uncertain Etiology (ISSUE) Investigators (2001). Mechanism of syncope in patients with isolated syncope and in patients with tilt-positive syncope. Circulation.

[21] Brignole M., Menozzi C., Del Rosso A., Costa S., Gaggioli G., Bottoni N., Bartoli P., Sutton R. (2000). New classification of haemodynamics of vasovagal syncope: Beyond the VASIS classification. Analysis of the pre-syncopal phase of the tilt test without and with nitroglycerin challenge. Vasovagal Syncope International Study. Europace.

[22] Baron-Esquivias G., Baron-Solis C., Ordonez A. (2019). Pacing for Patients Suffering From Cardioinhibitory Vasovagal Syncope Using the Closed-Loop System. Front. Cardiovasc. Med..

[23] Brignole M., Tomaino M., Gargaro A. (2017). Vasovagal syncope with asystole: The role of cardiac pacing. Clin. Auton. Res..

[24] Brignole M., Donateo P., Tomaino M., Massa R., Iori M., Beiras X., Moya A., Kus T., Deharo J.C., Giuli S. (2014). Benefit of pacemaker therapy in patients with presumed neurally mediated syncope and documented asystole is greater when tilt test is negative: An analysis from the third International Study on Syncope of Uncertain Etiology (ISSUE-3). Circ. Arrhythmia Electrophysiol..

[25] Baron-Esquivias G., Morillo C.A., Moya-Mitjans A., Martinez-Alday J., Ruiz-Granell R., Lacunza-Ruiz J., Garcia-Civera R., Gutierrez-Carretero E., Romero-Garrido R. (2017). Dual-Chamber Pacing with Closed Loop Stimulation in Recurrent Reflex Vasovagal Syncope: The SPAIN Study. J. Am. Coll. Cardiol..

[26] Rattanawong P., Riangwiwat T., Chongsathidkiet P., Vutthikraivit W., Limpruttidham N., Prasitlumkum N., Kanjanahattakij N., Kanitsoraphan C. (2018). Closed-looped stimulation cardiac pacing for recurrent vasovagal syncope: A systematic review and meta-analysis. J. Arrhythm.

[27] Palmisano P., Dell′Era G., Russo V., Zaccaria M., Mangia R., Bortnik M., De Vecchi F., Giubertoni A., Patti F., Magnani A. (2018). Effects of closed-loop stimulation vs. DDD pacing on haemodynamic variations and occurrence of syncope induced by head-up tilt test in older patients with refractory cardioinhibitory vasovagal syncope: The Tilt test-Induced REsponse in Closed-loop Stimulation multicentre, prospective, single blind, randomized study. Europace.

[28] Baron-Esquivias G., Moya-Mitjans A., Martinez-Alday J., Ruiz-Granell R., Lacunza-Ruiz J., Garcia-Civera R., Gutierrez-Carretero E., Romero-Garrido R., Morillo C.A. (2020). Impact of dual-chamber pacing with closed loop stimulation on quality of life in patients with recurrent reflex vasovagal syncope: Results of the SPAIN study. Europace.

[29] Gopinathannair R., Salgado B.C., Olshansky B. (2018). Pacing for Vasovagal Syncope. Arrhythmia Electrophysiol. Rev..

[30] Saal D.P., Thijs R.D., van Zwet E.W., Bootsma M., Brignole M., Benditt D.G., van Dijk J.G. (2017). Temporal Relationship of Asystole to Onset of Transient Loss of Consciousness in Tilt-Induced Reflex Syncope. JACC Clin. Electrophysiol..

[31] Brignole M., Tomaino M., Aerts A., Ammirati F., Ayala-Paredes F.A., Deharo J.C., Del Rosso A., Hamdan M.H., Lunati M., Moya A. (2017). Benefit of dual-chamber pacing with Closed Loop Stimulation in tilt-induced cardio-inhibitory reflex syncope (BIOSync trial): Study protocol for a randomized controlled trial. Trials.

[32] Akella K., Olshansky B., Lakkireddy D., Gopinathannair R. (2020). Pacing Therapies for Vasovagal Syncope. J. Atr. Fibrillation.

[33] Olshansky B. (2017). Vasovagal Syncope: To Pace or Not to Pace. J. Am. Coll. Cardiol..

[34] Virag N., Sutton R., Vetter R., Markowitz T., Erickson M. (2007). Prediction of vasovagal syncope from heart rate and blood pressure trend and variability: Experience in 1,155 patients. Heart Rhythm..

[35] Mallat Z., Vicaut E., Sangare A., Verschueren J., Fontaine G., Frank R. (1997). Prediction of head-up tilt test result by analysis of early heart rate variations. Circulation.

[36] Miranda C.M., Silva R. (2016). Analysis of Heart Rate Variability Before and During Tilt Test in Patients with Cardioinhibitory Vasovagal Syncope. Arq. Bras. Cardiol..

[37] Pachon M.J., Pachon M.E., Pachon C.T.C., Santillana P.T., Lobo T.J., Pachon M.J., Zerpa A.J., Cunha P.M., Higuti C., Ortencio F.A. (2020). Long-Term Evaluation of the Vagal Denervation by Cardioneuroablation Using Holter and Heart Rate Variability. Circ. Arrhythm Electrophysiol..

[38] Beitzke D., Wielandner A., Wollenweber T., Vraka C., Pichler V., Uyanik-Uenal K., Zuckermann A., Greiser A., Hacker M., Loewe C. (2019). Assessment of sympathetic reinnervation after cardiac transplantation using hybrid cardiac PET/MRI: A pilot study. J. Magn. Reson. Imaging.

[39] Pachon J.C., Pachon E.I., Cunha Pachon M.Z., Lobo T.J., Pachon J.C., Santillana T.G. (2011). Catheter ablation of severe neurally meditated reflex (neurocardiogenic or vasovagal) syncope: Cardioneuroablation long-term results. Europace.

[40] Pachon J.C., Pachon E.I., Pachon J.C., Lobo T.J., Pachon M.Z., Vargas R.N., Jatene A.D. (2005). "Cardioneuroablation"—New treatment for neurocardiogenic syncope, functional AV block and sinus dysfunction using catheter RF-ablation. Europace.

[41] Pachon M.E., Pachon-Mateos J.C., Higuti C., Santillana P.T., Lobo T., Pachon C., Pachon-Mateos J., Zerpa J., Ortencio F., Amarante R.C. (2020). Relation of Fractionated Atrial Potentials With the Vagal Innervation Evaluated by Extracardiac Vagal Stimulation During Cardioneuroablation. Circ. Arrhythm Electrophysiol..

[42] Akizuki H., Hashiguchi N. (2020). Heart rate variability in patients presenting with neurally mediated syncope in an emergency department. Am. J. Emerg. Med..

[43] Blendea D., McPherson C.A., Pop S., Anton F.P., Crisan S., Ruskin J.N. (2019). Isolated very low QRS voltage predicts response to tilt-table testing in patients with neurally mediated syncope. Pacing Clin. Electrophysiol..

[44] Madias J.E. (2008). Low QRS voltage and its causes. J. Electrocardiol..

[45] Brody D.A. (1956). A theoretical analysis of intracavitary blood mass influence on the heart-lead relationship. Circ. Res..

[46] Liu J.E., Hahn R.T., Stein K.M., Markowitz S.M., Okin P.M., Devereux R.B., Lerman B.B. (2000). Left ventricular geometry and function preceding neurally mediated syncope. Circulation.

[47] Shalev Y., Gal R., Tchou P.J., Anderson A.J., Avitall B., Akhtar M., Jazayeri M.R. (1991). Echocardiographic demonstration of decreased left ventricular dimensions and vigorous myocardial contraction during syncope induced by head-up tilt. J. Am. Coll. Cardiol..

[48] Blendea D., McPherson C.A., Pop S., Ruskin J.N. (2019). Isolated very low QRS voltage in the frontal leads predicts recurrence of neurally mediated syncope. Heart Rhythm.

[49] Grimm W., Degenhardt M., Hoffman J., Menz V., Wirths A., Maisch B. (1997). Syncope recurrence can better be predicted by history than by head-up tilt testing in untreated patients with suspected neurally mediated syncope. Eur. Heart J..

[50] Blendea D., Cimpeanu M., Jelnean M., Chiorescu R., Crisan S., Pop S. (2020). QRS voltage in precordial leads in patients with neurally mediated syncope. Eur. Heart J..

[51] Blendea D., Ruskin J.N., McPherson C.A. (2018). Vectorcardiographic QRS loop geometry in patients with neurally mediated syncope. Eur. Heart J..

[52] Blendea D., McPherson C., Pop S., Blachon A., Cimpeanu M., Ruskin J.N. (2020). Vectorcardiographic QRS loop geometry predicts recurrence of neurally mediated syncope. J. Am. Coll. Cardiol..

[53] Tentea C.-P., Chiorescu R., Crisan S., Pop S., Ruskin J.N., Blendea D. (2020). A Risk Score That Predicts Recurrence of Neurally Mediated Syncope Using Electrocardiographic and Vectorcardiographic Parameters. Circulation.

